# Comparison of Low Degree/High Degree and Zernike Expansions for Evaluating Simulation Outcomes After Customized Aspheric Laser Corrections

**DOI:** 10.1167/tvst.10.3.21

**Published:** 2021-03-23

**Authors:** Damien Gatinel, Jacques Malet, Laurent Dumas, Dimitri T. Azar

**Affiliations:** 1 Department of Anterior Segment and Refractive Surgery, Rothschild Ophthalmic Foundation Hospital, Paris, France; 2 Laboratoire de Mathématiques de Versailles, UVSQ, CNRS, Université Paris-Saclay, Versailles, France; 3 Department of Ophthalmology and Visual Sciences, University of Illinois at Chicago College of Medicine, Chicago, IL, USA

**Keywords:** wavefront, corneal topography, asphericity, Zernike, spherical aberration (SA)

## Abstract

**Purpose:**

The purpose of this study was to compare the low degree/high degree (LD/HD) and Zernike Expansion simulation outcomes evaluating the corneal wavefront changes after theoretical conventional and customized aspheric photorefractive ablations.

**Methods:**

Initial anterior corneal surface profiles were modeled as conic sections with pre-operative apical curvature, R0, and asphericity, Q0. Postoperative apical curvature, R1, was computed from intended defocus correction, D, diameter zone, S, and target postoperative asphericity, Q1. Coefficients of both Zernike and LD/HD polynomial expansions of the rotationally symmetrical corneal profile were computed using scalar products. We modeled different values of D, R0, Q0, S, and ΔQ = Q1 to Q0. The corresponding postoperative changes in defocus (Δz20 vs. Δg20), fourth order (Δz40 vs. Δg40) and sixth order (Δz60 vs. Δg60) Zernike and LD/HD spherical aberrations (SAs) were compared. In addition, retrospective clinical data and wavefront measurements were obtained from two examples of two patient eyes before and after corneal laser photoablation.

**Results:**

The z20, varied with both R0 and Q0, whereas the LD/HD defocus coefficient, g20, was relatively robust to changes in asphericity. Variations of apical curvature better correlated with defocus and ΔQ with SA coefficients in the LD/HD classification. The impact of ΔQ was null on g20 but induced significant linear variations in z20 and fourth order SA coefficients. LD/HD coefficients provided a good correlation with the visual performances of the operated eyes.

**Conclusions:**

Simulated variations in postoperative corneal profile and wavefront expansion using the LD/HD approach showed good correlations between defocus and asphericity variations with variations in corneal curvature and SA coefficients, respectively.

**Translational Relevance:**

The relevance of this study was to provide a clinically relevant alternative to Zernike polynomials for the interpretation of wavefront changes after customized aspheric corrections.

## Introduction

Modifying corneal asphericity is a method commonly used in excimer laser refractive surgery to alter the eye's spherical aberrations (SA).^1^^,^^2^ Reshaping the corneal contour by excimer laser photoablation is used to reduce the total ocular SAs while correcting the myopic or hyperopic defocus.^3^^,^^4^ Induction of negative SAs (Q_1_ < 0) creates a prolate or hyperprolate cornea, which may also serve to extend the depth of focus and through-focus visual acuity.^5^^–^^8^ Exploring the relationships between the changes in asphericity (ΔQ) and wavefront aberrations may be necessary to better assess the impact of the control of postoperative corneal asphericity on the low order (sphere/defocus and cylinder/astigmatism) and higher order aberrations of the corneal wavefront.^9^^–^^11^

To capture the impact of the custom aspheric laser profiles to the eye's total wavefront error, it is required to calculate the wavefront changes arising from the alteration of the anterior corneal surface's geometry. In the context of refractive surgery, the change in sphero-cylindrical error should ideally correspond to the variation of the lower order modes (defocus and astigmatism). Additionally, the variations in the corneal asphericity (ΔQ) should also ideally correspond to the variation of the higher order fourth and sixth radial order modes’ coefficients. Zernike modes, such as fourth and sixth SA (Z-HOAs Z_4_^0^ and Z_6_^0^), are containing some term in r^2^ (which corresponds to a defocus phase Z-LOA). For example, the Zernike polynomial defining “spherical aberration” includes both r4 and r2 terms and corresponds to the following wavefront error (WFE):

$$\begin{array}{c} WFE=c_{4}^{0}Z_{4}^{0}=c_{4}^{0}\sqrt{5}\left(6r^{4}-6r^{2}+1\right) \end{array}$$

As a consequence, the WFE within the central portion of the pupil is dominated by the opposite sign r^2^. Previous studies have shown that the visual impact of Zernike fourth and sixth order SA were mostly due to the lower order r^2^ term.^12^^,^^13^ This would suggest that using Zernike SA to analyze the wavefront changes caused by aspheric corrections may lead to difficulties in the interpretation of paraxial versus peripheral curvature changes, as it combines an r^2^ term with an r^4^ term. In addition, in the clinical setting, best visual acuity in subjective refractions for circular pupils are dominated by the central optics.^14^ This central defocus may contribute to the reduced accuracy of the prediction of spherocylindrical refractive error from the ocular low order wavefront component.^12^^,^^13^ As a corollary, the prediction of the visual impact of high degree aberrations after best spectacle correction is not realistic when modes, such as Zernike SA, for which the calculation of a point spreading function or a modulation transfer function includes the effect of the term in r^2^, whereas this would be largely neutralized by a defocus correction in glasses, to “flatten” the central part of the WFE.^12^^–^^15^ Seidel SA, on the other hand, describes a wavefront that is well focused centrally and either myopic (positive SA) or hyperopic (negative SA) at the pupil margins. Its analytical expression is limited to a term in r^4^. However, the Seidel class of aberrations is incomplete and not ortho-normalized over the circular pupil.

Motivated by the prospect of providing clinicians with an alternative set of aberration descriptors that would avoid mixing of lower degree and higher degree (LD/HD) terms within some of the Z-HOA modes, our group proposed an alternative polynomial decomposition method.^16^^,^^17^ This new non-Zernike expansion was generated to allow a clear cut separation between higher and lower order monomials within the higher and lower wavefront components. Importantly, the new higher order wavefront modes do not contain low (i.e. constant, linear, or quadratic) terms to provide a clinically relevant “low order wavefront error free” prediction of the visual impact of the higher order wavefront component. They are normalized and mutually orthogonal. The goal is to provide a clinically relevant “low order wavefront error free” prediction of the visual impact of the higher order wavefront component.

The purpose of this study was to compare this novel LD/HD method to the Zernike Polynomials method for evaluating corneal wavefront changes after custom aspheric corrections and estimate their clinical relevance from the intended corneal shape changes.

## Materials and Methods

### Determination of Corneal Wavefront Profile Changes After Conventional and Customized Corrections Using Zernike and LD/HD Polynomial Expansions

**Figure 1.**
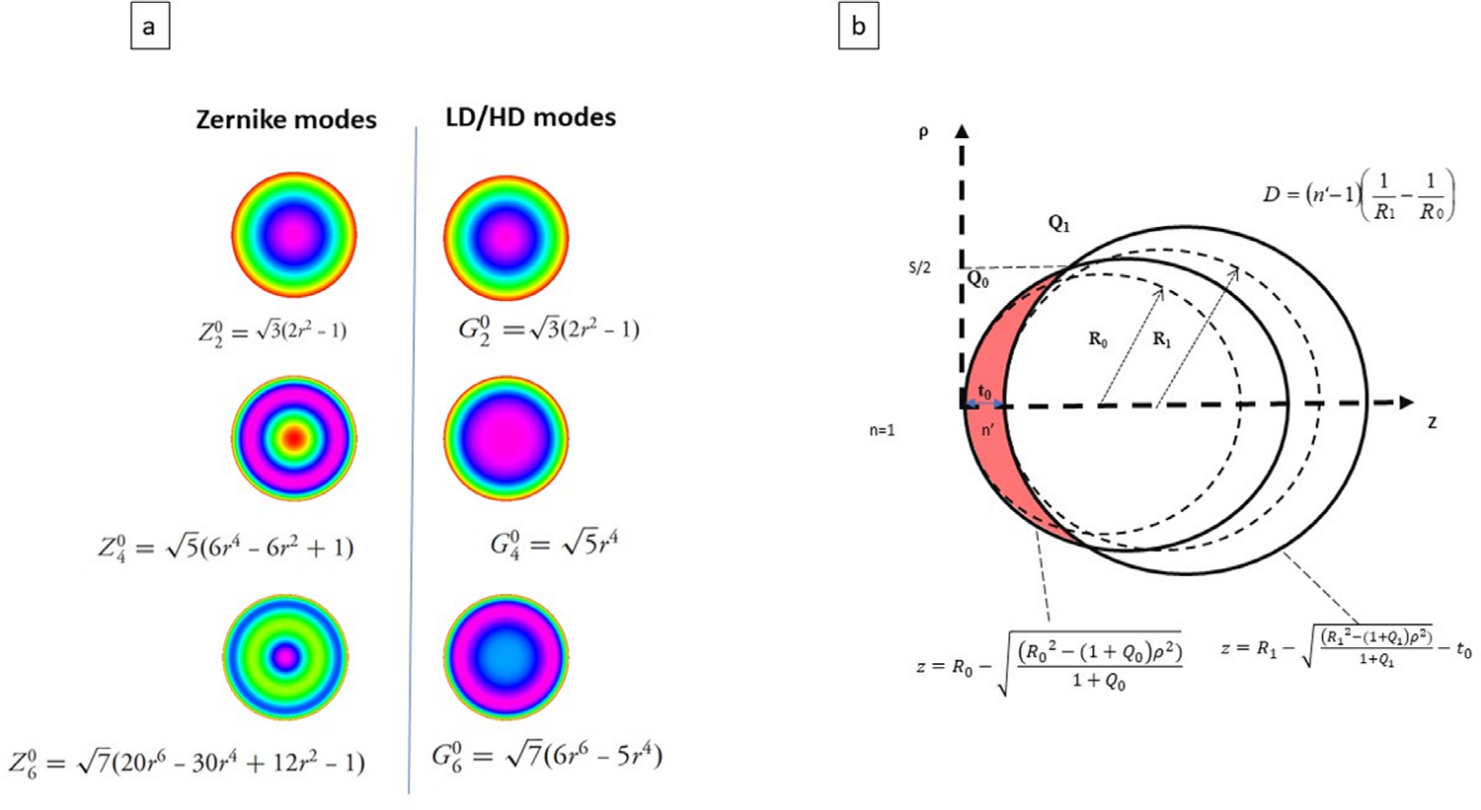
(**a**) Rotationally symmetrical modes of the Zernike and LD/HD modes of maximum radial order six and their analytical expression. (**b**) Determination of the aspheric profile of ablation for defocus correction and control of the corneal asphericity over an optical zone of S diameter (outlined in *red*, custom myopic ablation). R_0_ and R_1_, Q_0_ and Q_1_ correspond to the apical radii and asphericities of the initial and final conic sections modeling the corneal surfaces, respectively. The n’ is the refractive index of the corneal stroma, and t_0_ is the depth of ablation at the center of the optical zone.

We modeled the pre-operative and postoperative corneal surface profile as conic sections. We limited our analysis to rotationally symmetrical aberrations, and did not take into consideration the effect of the transition zones (Figs. 1a, 1b).

By using scalar products, the approximation of the rotationally symmetrical corneal surface, whose profile is a conic section, can be converted in a rotationally invariant Zernike polynomial expansion (m = 0) up to the sixth radial order (*n* = 6) over a zone of diameter S.^18^ This allows us to obtain the values of the coefficients c_2_^0^, c_4_^0^, and c_2_^0^ of the Z_2_^0^, Z_4_^0^, and Z_6_^0^ polynomial modes, respectively.

The theoretical value of the postoperative apical radius of curvature, R_1_, was computed using a paraxial formula from the value of the preoperative apical radius R_0_ and the distance defocus, D, at the corneal plane. The pre and postoperative asphericity values were adjusted to conform to the characteristics (conventional or customized) of the ablation profile.

The values of the coefficients c_2_^0^, c_4_^0^, and c_6_^0^ of the rotationally invariant Z_2_^0^, Z_4_^0^, and Z_6_^0^ Zernike polynomials corresponding to the preoperative (R_0_ and Q_0_) and postoperative (R_1_ and Q_1_) spherical corneal profiles were obtained by using scalar products on a zone of diameter S. Using the LD/LH method described previously, the coefficients g_2_^0^, g_4_^0^, and g_6_^0^ weighting the new polynomials G_2_^0^, G_4_^0^, and G_6_^0^ can be directly computed analytically from the coefficients c_2_^0^, c_4_^0^, and c_6_^0^ weighting a Zernike expansion for the same fit order.^16^

The variations in the rotationally symmetrical corneal Zernike and LD/HD wavefront coefficients, caused by the subtraction of the ablation profile from the corneal surface were computed as the difference between the final postoperative, z_n_^0^f or g_n_^0^f and the initial preoperative, z_n_^0^i or g_n_^0^i, values multiplied by the change in the refractive index (n’-n) from the air (n = 1) to the corneal stroma (n’ = 1.376) ^19^:

$$\begin{array}{c} \Delta z/g_{n}^{0}=\left(z/g_{n}^{0}f-z/g_{n}^{0}i\right)\times 0.376. \end{array}$$

For normalized Zernike and LD/HD second degree coefficients, the dioptric spherical equivalent (SE) of defocus coefficients (or variations of) was computed as:

$$\begin{array}{c} SE=\frac{4\sqrt{3}}{{\left(S/2\right)}^{2}}\;c{\;}_{2}^{\;0} \end{array}$$

where c_2_^0^ is the (root mean square [RMS]) amplitude of the Z_2_^0^ Zernike or G_2_^0^ LD/HD modes in micrometers.

### Determination of Corneal Wavefront Profile Changes After Conventional Non-Custom Spherically Based Profiles of Ablation Using Zernike and LD/HD Polynomial Expansions

To investigate the theoretical change on the corneal wavefront of conventional noncustom spherically based corneal profiles ranging from myopic to hyperopic corrections (−10 D to +6 D), both the initial and final surface profiles’ asphericity were set to 0 (Q_0_ = Q_1_ = 0). Zernike and LD/HD coefficients variation corresponding to the corresponding optical path change were computed. The impact of the optical zone on the Zernike and LD/HD coefficients and predicted SE changes was evaluated for diameter comprised between 5.5 and 8 mm for a −6 D correction.

### Determination of Corneal Wavefront Profile Changes After Customized Aspheric Corrections Using Zernike and LD/HD Polynomial Expansions

The theoretical variation in the corneal wavefront coefficients caused by the change in the value of the apical radius and asphericity of the corneal profile were computed for custom aspheric profiles of ablation covering three clinical scenarios: (1) reducing excessive corneal oblateness (Q_0_ = +0.5) after myopic surgery, (2) reducing excessive corneal prolateness (Q_0_ = −0.8) after hyperopic surgery, and (3) introducing a fixed amount of Zernike negative SA (Δz_4_^0^ = −0.4 microns) while inducing myopic refraction for the correction of presbyopia in patients with hyperopia.

**Table.** Effect of Hyperopic Correction Aimed at Changing the Amount of Fourth Order Zernike Spherical Aberration (Δz_4_^0^ = −0.40 Microns) on the Zernike and LD/HD Defocus Coefficients and Computed Change in Spherical Equivalent (ΔSE)

Planned Correction, D	Q1	R_1_, mm	Δz_2_^0^, Microns	Δg_2_^0^, Microns	ΔSE Zernike, D	ΔSE LD/HD, D
+2.00	−0.82	7.49	1.12	2.59	+0.86	+2.00
+4.00	−0.84	7.20	3.72	5.19	+2.86	+4.00
+6.00	−0.86	6.94	6.32	7.78	+4.86	+6.00

In addition to our theoretical modeling, two clinical examples are presented in the Appendix.

## Results

### Determination of Corneal Wavefront Profile Changes After Conventional Noncustom Spherically Based Profiles of Ablation Using Zernike and LD/HD Polynomial Expansions

#### Influence of the Magnitude of the Correction

**Figure 2.**
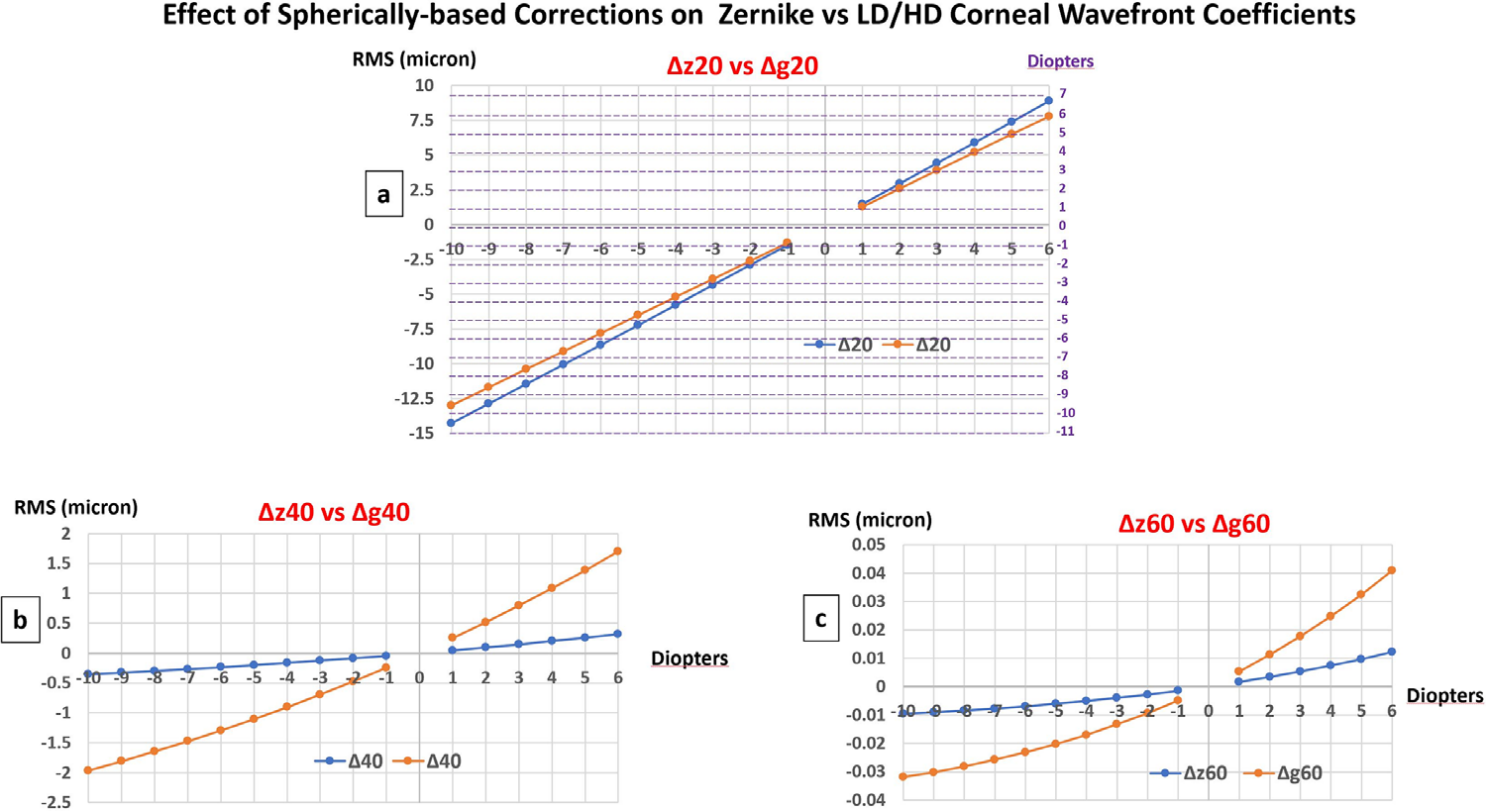
Effect of noncustom spherically based profile ablation on the variations of Zernike versus LD/HD corneal wavefront coefficients for spherical corrections comprised between −10 D and +6 D.

The values of the Z-LOA defocus coefficients were slightly lower for myopic corrections and slightly higher for hyperopic corrections than the corresponding LD/HD defocus coefficients values (Fig. 2a). The computed SE was equal to the intended paraxial corrections in Diopters for the LD/HD defocus coefficients (g_2_^0^), whereas the equivalent dioptric defocus computed from the z_2_^0^ defocus coefficients exceeded the magnitude of the intended correction. The maximum difference was an excess of 1.0 D myopic power for the −10 D correction and of 0.86 D hyperopic power for the +6 D correction. The variations of the fourth and sixth (higher order) coefficients were similar in trend but different in magnitude between the Zernike and LD/HD coefficients (Figs. 2b, 2c). Theses variations were negative (increased negative SA) for myopic corrections, and positive (increased positive SA) for hyperopic corrections.

#### Influence of the Optical Zone Diameter, S

**Figure 3.**
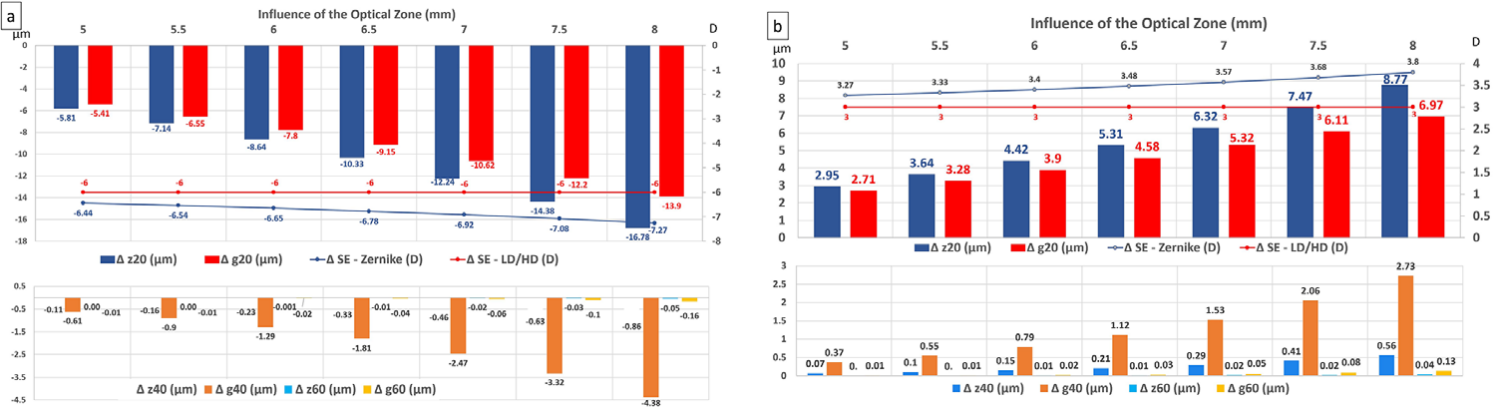
Influence of the optical zone diameter on the rotationally symmetrical coefficients of the Zernike and LD/HD expansions and the change in spherical equivalent (SE) computed from the values of the z_2_^0^ versus g_2_^0^ coefficients. (**a**) Myopic correction (−6 D). (**b**) Hyperopic correction (+3 D).

Figures 3a and 3b shows the theoretical influence of the planned optical zone diameter, S, ranging from 5.00 to 8.00 mm, on the values of the rotationally symmetrical coefficients of the Zernike and LD/HD classifications for noncustom spherically based myopic (−6 D) and hyperopic (+3 D) corrections. The SE is systematically overestimated by the calculation carried out with the coefficient z_2_^0^, by an amount that increases with the diameter of the optical zone. The fourth and sixth order Zernike and LD/HD coefficients increased proportionally to the change in diameter of the optical zone raised to power four and to power six, respectively. The changes in SE computed from the g_2_^0^ coefficient matches the intended paraxial correction D and remains unaffected by the variations in the OZ diameter.

### Determination of Corneal Wavefront Profile Changes After Customized Aspheric Corrections Using Zernike and LD/HD Polynomial Expansions

#### Custom Ablations for Correcting Oblate Corneas With Unchanged Apical Radius of Curvature

**Figure 4.**
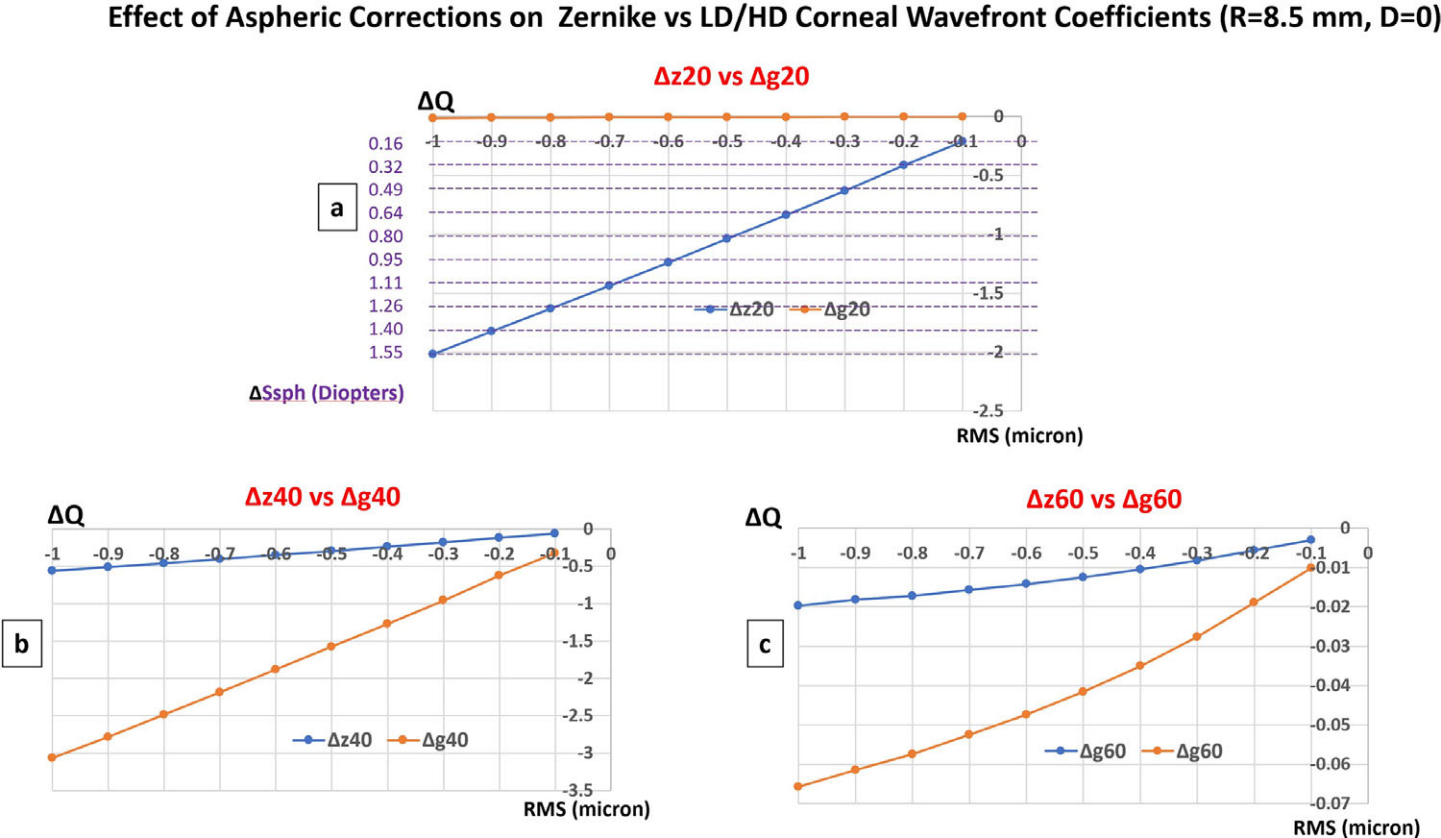
Impact of custom aspheric correction aimed at increasing corneal prolateness on Zernike versus LD/HD rotationally invariant coefficients of the corneal wavefront on a 6 mm zone.

In this scenario, the Zernike and LD/HD expansions were compared for custom aspheric profiles delivered on a flat oblate corneal surface (Q_0_ = +0.5, R_0_ = 8.5 mm) targeting different variations of the corneal asphericity from ΔQ = −0.1 to ΔQ = −1 by 0.1 steps while leaving the paraxial curvature unchanged (D = 0, R_0_ = R_1_; see Fig. 4a). Whereas the g_2_^0^ coefficients remain null regardless of the value of the target asphericity, a change of z_2_^0^ coefficient toward more negative values is observed, corresponding to a dioptric defocus equivalent change in the direction of less myopia/more hyperopia and comprised between ΔD = +0.16 D (ΔQ = −0.1, Δz_2_^0^ = −0.21 microns) and ΔD = +1.55 D (ΔQ = −1, Δz_2_^0^ = −2 microns). The larger the change toward less oblate/more prolate asphericity, the larger the change in the fourth and sixth order Zernike and LD/HD SA coefficients toward more negative values see Figures 4b, 4c.

#### Custom Ablations for Correcting Hyperprolate Corneas With Unchanged Apical Radius of Curvature

**Figure 5.**
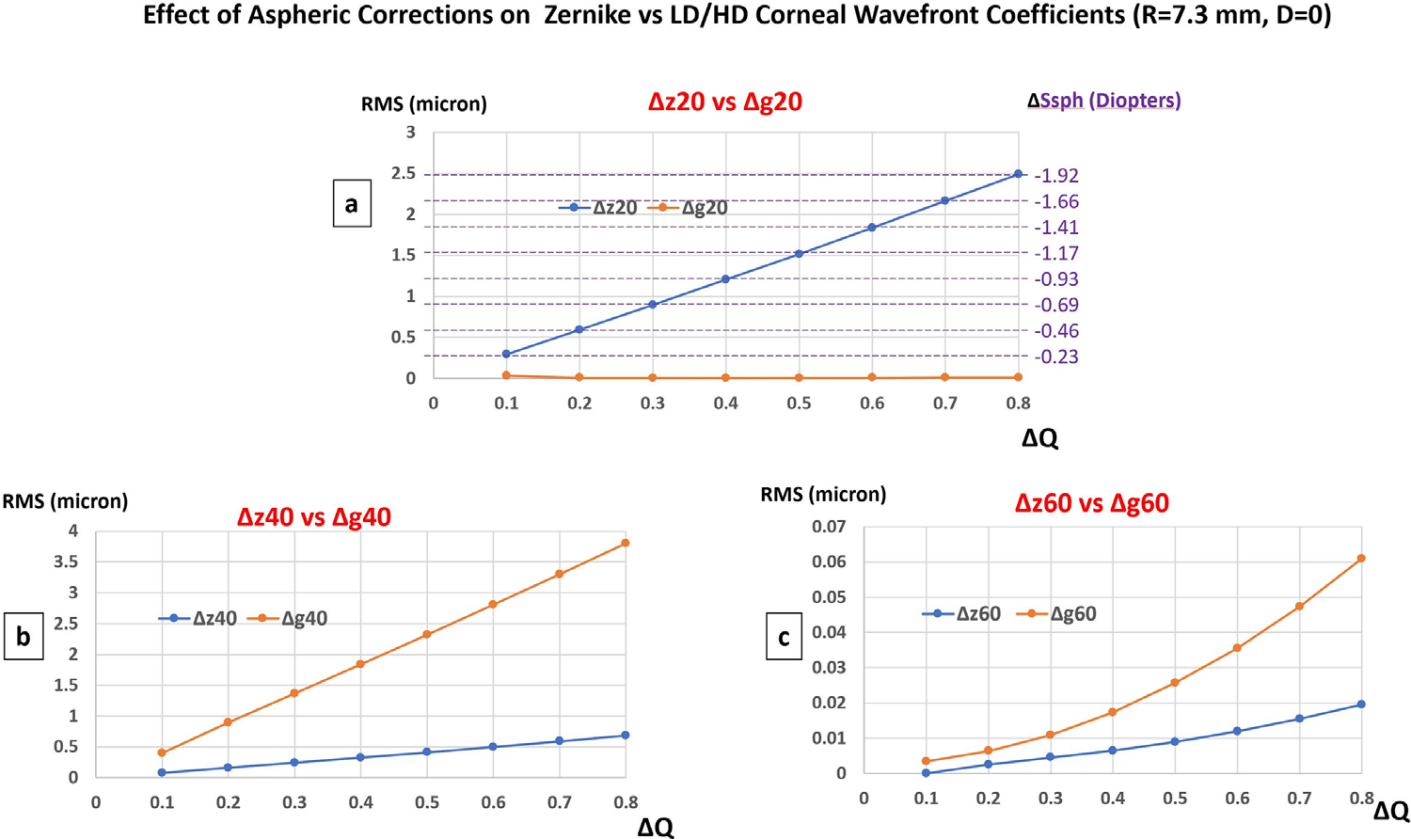
Impact of custom aspheric correction aimed at increasing corneal oblateness on Zernike versus LD/HD rotationally invariant coefficients of the corneal wavefront on a 6 mm zone.

In this scenario, the Zernike and LD/HD expansions were compared for custom aspheric profiles delivered on a steep prolate corneal surface (Q = −0.8, R0 = 7.3 mm) targeting positive variations of the corneal asphericity comprised between ΔQ = +0.1 and ΔQ = +0.8 by 0.1 steps while leaving the paraxial curvature unchanged (D = 0, R_0_ = R_1_ = 0; see Fig. 5a). The change in z_2_^0^ coefficients values correspond to a defocus change in the direction of less hyperopia/more myopia and comprised between ΔD = −0.23 D (ΔQ = +0.1, Δz_2_^0^ = +0.29 microns) and ΔD = −1.92 D (ΔQ = +0.8, Δz_2_^0^ = +2.49 microns). The larger the change toward less prolate asphericity, the larger the change in the fourth and sixth order Zernike and LD/HD SA coefficients toward more positive values see Figures 5b, 5c.

#### Custom Ablations for Presbyopia

In this third scenario, we have used the concepts presented in previous theoretical and clinical work concerning the achievement of compensation for presbyopia in hyperopes customized correction aimed at inducing a paraxial myopic refraction while changing the amount of corneal fourth order Zernike SA of an amount Δz_4_^0^ = −0.40 microns using a customized aspheric ablation profile to increase corneal prolateness. For hyperopic corrections and large intended changes in SA (Δz_4_^0^ = −0.4 µm, equivalent to Δg_4_^0^ = −2.27 microns), the required change in asphericity can be computed depending on the preoperative corneal asphericity and planned paraxial defocus correction.

The Table shows the variables, such as target apical radius of curvature (R_1_) and asphericity (Q_1_), along with the theoretical Zernike and LD/HD wavefront expansions caused by such custom profile for various defocus corrections (+2 D, +4 D, and +6 D) applied on a preoperative corneal profile (R_0_ = 7.8 mm, Q_0_ = −0.2, 6 mm zone). The dioptric change estimated from the variation in the z_2_^0^ coefficient underestimates the planned defocus change by an amount of +1.24 D.

## Discussion

The LD/HD expansion method based on new polynomial functions to distinguish between low and high degree aberrations has been proposed recently.^16^^,^^17^ The decomposition of the wavefront in our new basis requires new coefficients, which are obtainable from Zernike expansion after collection of low order terms. It allows to express two components for the total wavefront by grouping the modes according to their radial order. The LD component of the wavefront can be described with an expansion of weighted low order modes of the same analytical structure as their Zernike counterparts. It is expressed as an expansion of the new higher order modes, which are all devoid of lower order terms. Studies have shown that marginal optics have little or no impact on the full pupil spherical refraction,^12^^–^^14^^,^^20^^–^^22^ which suggests that high spatial frequency refractions achieve a near paraxial focus. In the presence of SA, authors have shown that visual acuity was better with paraxial focus than with the defocus that minimized RMS.^23^ Although not exactly equivalent to paraxial curvature matching of the wavefront, the spherical equivalent error appears to be dominated by the near paraxial optics.^15^ This novel polynomial decomposition basis approach, in which the low order wavefront component is equal to the paraxial curvature matching of the wavefront map, was used to improve the prediction of subjective refraction from wavefront aberrometry data processed with machine learning algorithms and better apprehend the impact of higher order wavefront phase errors.^24^ Machine learning models were significantly better than the paraxial matching method; however, it showed that G_2_^0^ (paraxial defocus) was by far the most influential feature to predict the SE value, with G_4_^0^ (primary SA) being the second most important feature. These data suggest that the use of paraxial curvature matching for defining the low order component is clinically more relevant for some clinical applications. The use of high-degree modes whose analytical expression is devoid of high degree has the advantage of removing the ambiguity associated with the visual impact of degree 2 monomials.

### Determination of Corneal Wavefront Profile Changes After Conventional Noncustom Spherically Based Profiles of Ablation Using Zernike and LD/HD Polynomial Expansions

The differences in fitting methods have also important consequences for characterizing the wavefront changes induced by the conventional and aspheric ablation profiles in refractive surgery. The dioptric equivalent change resulting from the variation of the second degree coefficients should ideally match the planned spherical correction whereas the modulation of the corneal asphericity be reflected in the variation of fourth and sixth order coefficients only. The predicted equivalent dioptric variation from Zernike and LD/HD respective defocus coefficient (z_2_^0^ vs. g_2_^0^) is shown both spherically based and customized aspheric profiles of ablation (Figs. 2a, 4a, 5a).

For noncustom spherically based corrections, the magnitude of the intended change in refractive sphere is slightly overestimated when computed from the z_2_^0^ coefficient, whose value is affected by the need to compensate for the r^2^ terms of the z_4_^0^ and z_6_^0^ modes (see Fig. 2a). This difference increases with the diameter of the optical zone. Spherical myopic corrections introduce an amount of negative fourth and sixth order Zernike and LD/HD aberrations (Figs. 2b, 2c). These are caused by the conjunction of the flattening of the corneal surface and the increase in prolateness predicted after spherically based profiles of ablation for initially prolate corneas.^25^

### Determination of Corneal Wavefront Profile Changes After Customized Corrections Using Zernike and LD/HD Polynomial Expansions

#### Custom Ablations for Correcting Oblate Corneas With Unchanged Apical Radius of Curvature

A theoretical variation of the coefficient z_2_^0^ occurs with the ablation profiles aiming at the induction of a simple modification of the asphericity without modification of the apical power (zero dioptric correction). The higher the change in asphericity (ΔQ), the higher the change in z_2_^0^, and therefore the predicted variation in dioptric spherical equivalent. As an example, a change of ΔQ = −0.8 (increased prolateness and reduced oblateness) causes a theoretical dioptric defocus variation of the Z-LOA wavefront close to of 1.25 D (myopic shift). Conversely, using the LD/HD decomposition method, the variation of the g_2_^0^ coefficient is null (Figs. 4a, 5a), as should be theoretically expected (D = 0). This discrepancy can cause a bias in interpretation and lead the surgeon inspecting the planned change in the Zernike coefficients to believe that a programmed ablation profile will modulate the planned correction of some amount of defocus whereas this one is nothing other than an artifact linked to necessity to balance the second degree radial term in the Z_4_^0^ and Z_6_^0^ modes. These spurious interactions are even more pronounced for custom ablations aimed at reducing the prolateness of a steep cornea (the resulting change in the z_2_^0^ coefficient predicts a hyperopic shift of 1.90 D for ΔQ = +0.8).

The interactions between the low order terms within the rotationally Z-HOA polynomials also reduce the relevance of the prediction of defocus change by the coefficient z_2_^0^ for custom aspheric profiles aimed at inducing myopic defocus and inducing certain amounts of SA to compensate for presbyopia, as shown in the Table. The aim is to create a more curved surface in the central zone and a flatter one in the peripheral zone so that for small pupils the vision would be dominated by this central zone, improving near vision, whereas for large pupils, the vision would be dominated by the peripheral corneal zone, providing acceptable distance vision.

#### Custom Ablations for Correcting Hyperprolate Corneas With Unchanged Apical Radius of Curvature

The change in the spherical equivalent is underestimated in the Zernike expansion of the expected corneal wavefront changes by an amount close to 1.25 D, due to the induction of a Δz_2_^0^ change of negative sign to compensate for the quadratic component included in the Z_4_^0^ mode, which results from the modulation of the corneal asphericity toward increased prolateness. This amount is subtracted to the positive change in Δz_2_^0^, which is induced by the positive spherical correction and reduces the net apparent defocus variation. In our simulations, the difference between the z_2_^0^ and g_2_^0^ coefficient is roughly equal to approximately 15^0.5^ or 3.9 times Δz_4_^0^, as expected from the analytical structure of the Z_4_^0^ mode. Meanwhile, the magnitude of the variation of fourth order LD/HD SA is roughly six times that of the Zernike SA (approximately Δg_4_^0^ to 6xΔz_4_^0^).

Varifocal ablations (SupraCor; Technolas Perfect Vision GmbH, Munich, Germany) are intended to induce negative SAs to increase multifocality and benefit near and far vision simultaneously. When emmetropia is the target refraction, Taneri et al. have shown that varifocal excimer laser ablation profile yield no additional benefit compared to monofocal ablations in hyperopic presbyopic laser-assisted in situ keratomileusis (LASIK), confirming the need for a myopic target to allow an effective multifocality on near vision.^26^ Although the exact characteristics of the delivered profile is proprietary, the increase of negative SA requires an increased corneal prolateness. The interpretation of the changes in defocus of the corneal wavefront using Zernike reconstruction following Varifocal ablation should be cautious, as the shift toward increased negative asphericity may result in a significant negative variation of the z_2_^0^ wavefront coefficient (see Fig. 5a).

#### Custom Ablations for Presbyopia

Some authors have reported the outcomes of a central presbyLASIK with corneal asphericity modulation by Q-factor modification of the F-CAT program in the nondominant eye.^27^^–^^29^ Some authors^28^^,^^29^ acknowledge that a re-adjustment of target refraction by myopization was required to compensate for the defocusing induced by Q-factor modification. They attributed the apparent hyperopic shift (increase in negative Zernike defocus) predicted from the treatment planner to the change in corneal asphericity, which in fact results from the interactions with the Zernike SA discussed in the present study. The negative SA induction through multifocal ablation profile based on increased corneal prolateness requires a myopic paraxial refraction to reach better near vision while improving distance vision over classic monovision.^7^^,^^30^ Indeed, in an eye paraxially emmetropic, increased corneal prolateness inducing negative SA would result in a hyperopic shift for nonparaxial rays, which would not be useful for near vision.

The presence of low-level terms in Zernike's SA mode also has consequences in establishing personalized topographic corrections, especially those planned with the “Contoura” system. Some surgeons recommend programming a defocus correction, which will make the Zernike defocus coefficient z_2_^0^ (commonly designated as C4 in the single index notation used by the laser manufacturer) equal to the value of the fourth order SA coefficient z_4_^0^ (designated as C12).^31^

Although we have limited our analysis to rotationally symmetrical coefficients, it is expected that similar interactions between high and low order modes would occur for some pairs of Zernike modes, such as coma Z_3_^±1^ and tilt Z_1_^±1^, or secondary astigmatism Z_4_^±2^ and primary astigmatism Z_2_^±2^. These interactions could explain the discrepancies between anterior corneal astigmatism and refractive astigmatism when analyzed through Zernike polynomial decompositions in the context of topography-guided ablations.^32^ Further studies are required to evaluate the potential benefit of the LD/HD decomposition method on the nonrotationally invariant component of the wavefront. We limited our calculations to the corneal plane: the net contribution of the change in SA of corneal origin to the ocular wavefront may be slightly different and would take into account the distance and possible decentration from the entrance pupil to the corneal plane. Our static subtraction shape model neglected the impact of some physical constraints, such as the “cosine effect” along with that of the biomechanical and wound healing response. However, we postulate that improving the relevance of wavefront interpretation may help to better segregate between the impact of multiple variables on the measured outcomes in clinical practice.

### Clinical Cases (Appendix)

We found acceptable agreement between the predicted and measured wavefront second and fourth order rotationally symmetrical coefficients, despite the assumptions of our model, which was limited to paraxial and aspheric changes at anterior corneal surfaces modeled as conic sections. The increased discrepancy for the sixth order coefficients reflects the limits of this model limited to two descriptors: apical radius and asphericity.^33^^,^^34^ Separate analysis of radius and asphericity incorrectly estimates the statistical significance of the changes in the ocular surfaces, and a new representation is there proposed.

Nevertheless, we observed the same trends predicted by our theoretical simulations regarding the variations in defocus and SE, which were underestimated in the Zernike decomposition because of the interaction between the second order monomials parsed in the low and high order modes.

**Figure A2.**
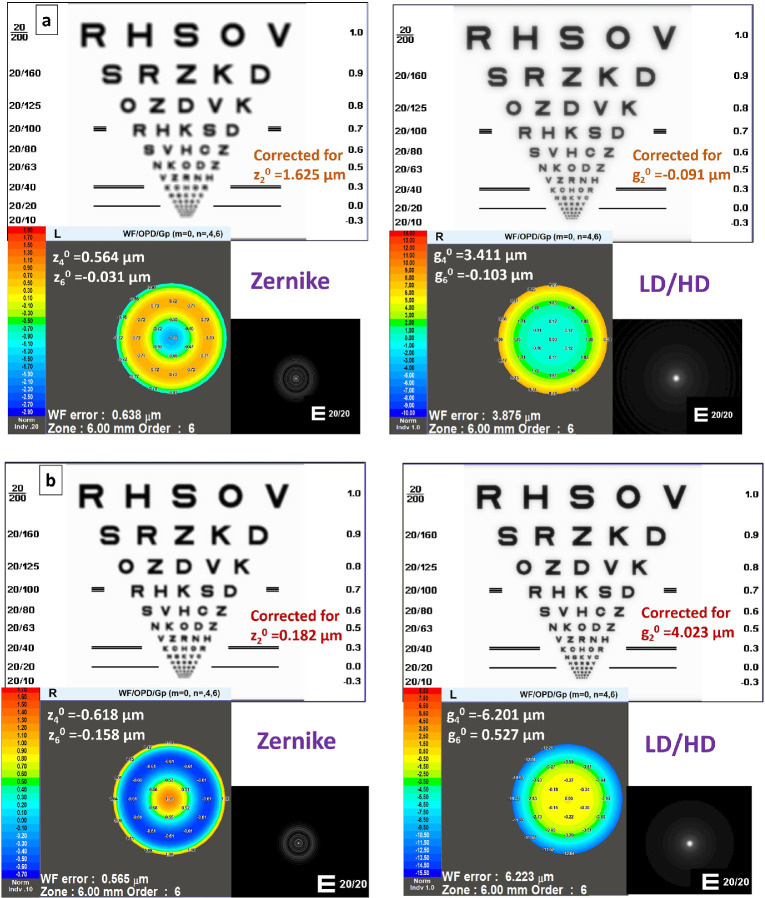
Retinal image simulation of a Snellen chart (*top*) for the higher order component of the wavefront (*bottom left*) and its corresponding PSF (*bottom right*). Left : Zernike decomposition. Right : LD/HD decomposition. (**a**) Case 1. (**b**) Case 2.

In case 1, the eye is emmetropic, but the predicted SE from the z_2_^0^ is slightly myopic, which contradicts the 20/15 uncorrected visual acuity. This positive Zernike defocus coefficient correlates with the need for compensating for the negative lower order term in r^2^ embedded within the analytical expression of the Z_4_^0^ mode. There is negligible defocus in the LD/HD decomposition. When the higher order component of the Zernike decomposition remains uncorrected, the Snellen chart simulations suggest an exaggerated visual blur, which is mainly caused by the quadratic central wavefront error (r^2^ monomial) that is well visible on the representation of the 6 mm pupil Zernike higher order wavefronts, whereas the HD envelopes are flatter paraxially (Fig. A2). When computed from the LD/HD decomposition, the simulated Snellen chart retinal image for the uncorrected higher order component (HD) is in line with the eye's visual performance.

Various retinal image quality metrics have been shown to account for the influence of both high and low order aberrations terms on best focus.^35^^–^^37^ Generally, the defocus term that maximizes such metrics is, to date, the best predictor of subjective spherical error. Visual acuity is determined primarily by the WFE in the central portion of the pupil, and it is plausible that the r^2^ term generating spherical defocus-like wavefronts in the pupil center for a wavefront dominated by Zernike SA, such as in case 2 would be flattened or canceled by the defocus of the best spectacle correction (the theoretical amount of defocus required to cancel the r^2^ term within Z40 is $z_{2}^{0}=\sqrt{15}z_{4}^{0}$). This explains the discrepancy between the value of z_2_^0^ and that of g_2_^0^ and SE in the case 2. Using the LD/HD decomposition results in the decoupling of the r^2^ term embedded in the Z_4_^0^ mode and unmask the low order paraxial WFE. In addition, as in case 1, that r^2^ term reduces the quality of the predicted retinal image of the higher order Zernike WFE.

Alternative representations to the Zernike polynomials have been used in visual science, such as Fourier, Seidel ot Taylor series.^38^^,^^39^ The LD/HD decomposition method is intended to supplement, rather than replace, the Zernike polynomials. It is aimed to provide clinicians with an alternative set of weighted higher order modes over circular pupils that would reflect the lower order (i.e. correctable with spectacles) versus higher order contribution to the ocular wavefront.

Although some of the Zernike polynomials aspects and drawbacks are not new and have been discussed previously,^40^^–^^43^ they play important roles in various optics branches, such as beam optics and the study of single- and multiple-circular aperture optical systems that are affected by atmosphere turbulence, or optical metrology for surface and transmitted wavefront representation.^44^ Their performance on elliptical pupils has been studied.^45^^,^^46^ Their full orthogonality confers some advantages, such as the robustness of coefficients to the truncation of an expansion, and the aberration balancing property, which leads to minimal variance (48). This property is conferred to an aberration by the mix of aberrations of lower order; we posit that this leads to potential interpretation problems in some visual optics applications where a clearer cut between the paraxially dominated low and marginally dominated higher order WFE is needed, such as discussed in this paper. As there is a univocal correspondence between the coefficients in both bases, the differences between them represent an interpretation of the respective role of low and/or higher order modes, but not an improvement in terms of quality of fitting or residuals minimization. Because of the lack of orthogonality between the LD and HD components, it is not possible to compute RMS values including a mixture of both LD and HD coefficients, and the RMS does not always correspond to the standard deviation of the WFE. In ophthalmic optics, clinical interpretation considers lower order aberrations and HOAs separately. The impact of the low order component of the wavefront is usually expressed separately and/or through the classic expression of spherocylindrical refraction into diopters, whereas the main contribution of wavefront analysis is to provide the higher order RMS value by concentrating only on the coefficients of the higher order polynomials. To satisfy the constraint of orthogonality between the new high degree modes, the presence of terms of variable degree (but always strictly greater than or equal to *n* = 3) is necessary from the fifth odd degree (they contain monomials of degree 3) and sixth degree even (they contain monomials of degree 4). The interpretation of the coefficients weighting the aberrations of radial degree s3 and 4 must be cautious in clinical situations that involve an elevation of the aberrations with higher radial degree.

### Conclusion

In conclusion, we have established the theoretical relationships between the change in corneal shape parameters and the resultant variation of the Zernike and LD/HD coefficients for rotationally symmetrical aberrations. Although a variation in asphericity without modification of the apical curvature of the cornea should ideally lead to an isolated modulation of the SA coefficients of the corneal wavefront, we observed that it caused a significant variation in the defocus coefficient of the Zernike classification. These theoretical predictions were echoed in the characterization of the wavefront changes of two patient eyes after custom aspheric corneal photoablation. In such clinical application, accurate distinction between lower and higher wavefront components is mandatory to accurately predict spectacle refraction and accurate retinal image metrics. We demonstrated the following additional advantages of the LD/HD method as compared to Zernike: whereas the Zernike defocus coefficient varied with both apical radius and asphericity and these changes increased with the diameter of the treatment zone, the LD/HD defocus coefficient was robust to changes in asphericity. Overall, the variations of the apical corneal curvature are naturally correlated with the variations of paraxial defocus and the variations of asphericity better correlated with the variations of the SA coefficients in the LD/HD classification than in that of Z-HOA. The impact of the induced postoperative asphericity on the defocus coefficients is negligible with the LD/HD decomposition. This method allows the clinician to link the modifications of the paraxial curvature with the variations of defocus, and the modifications of the asphericity with the variations of the wavefront coefficients assigned to the high degree modes with symmetry of revolution. These results suggest that this approach is more relevant to estimate the theoretical modifications induced at the level of the corneal wavefront by a personalized aspheric ablation profile.

## Figures and Tables

**Figure A1.**
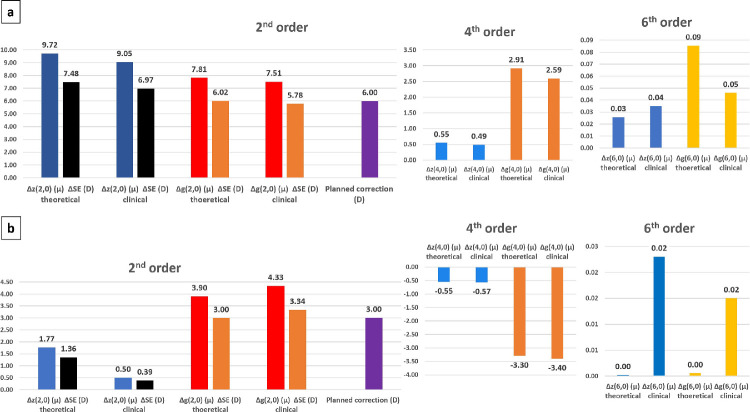
Comparison between the planned correction and the predicted and measured second, fourth, and sixth order coefficients of Zernike versus LD/HD rotationally symmetrical modes over a 6 mm pupil for the eyes presented in case 1 (**a**) and in case 2 (**b**).

## Appendix: Clinical Examples

The anterior corneal topography and ocular wavefront data obtained from two patient eyes operated with custom aspheric laser-assisted in situ keratomileusis (LASIK) were studied retrospectively. Because the analysis of the corneal wavefront with the OPDscan is proprietary and based on assumptions that may reduce the relevance of pre to postoperative low order coefficients comparisons, the total (ocular) wavefronts acquired in the same conditions pre and postoperatively were considered for our analysis. These eyes were selected because the treatment strategy incurred a planned aspheric variation and significant postoperative variations in the magnitude of defocus and spherical aberration. The surgeries were performed using a femtosecond laser for the creation of the LASIK flap (FS200; Alcon Wavelight) and an excimer laser for the refractive correction (EC500; Alcon Wavelight, Fort Worth, TX, USA) using the custom-Q option for treatment planning. From pre and 1-month postoperative topo-aberrometric measurements (OPDscan III, Nidek, Gammagori, Japan), the mean values of the 1 mm central keratometry and the anterior corneal asphericity (Q) provided by the topographer were collected and used as a surrogate of the apical curvature and asphericity of the corneal profile assimilated to a conical section. Using these values, the theoretical values corresponding to the variations of the coefficients assigned to the aberrations with rotational symmetry up to the sixth radial degree were calculated in the Zernike classification and then converted in their corresponding low degree/high degree (LD/HD) values. They were compared with the pre versus postoperative variations actually measured within the ocular wavefront by the aberrometer. The variations in the low order wavefront components of the Zernike and LD/HD decompositions were compared to the planned spherical equivalent correction. The postoperative values of the z_2_^0^ and g_2_^0^ coefficients were converted in the dioptric defocus and compared to the postoperative spherical equivalent (SE) of the analyzed eyes. The Snellen chart retinal image simulations were obtained via convolutional techniques from the point spread function (PSF) computed for the Zernike versus LD/HD higher order components. Low order corrected retinal image simulations were generated and compared between the Zernike and LD/HD wavefront reconstructions.

### Case 1

A custom aspheric LASIK was performed to correct for a hyperopic SE of +6 D with an intended variation in asphericity of ΔQ = +0.3 to anticipate an increase in negative spherical aberration.^20^ The mean values of the pre and postoperative 1 mm central keratometry and the anterior corneal asphericities were: R_0_ = 7.75 mm, R_1_ = 6.89 mm, Q_0_ = −0.02, and Q_1_ = 0.18. Figure A1 allows to compare the theoretical versus measured variations for the second, fourth, and sixth order coefficients of the corresponding Zernike versus LD/HD modes (6 mm pupil). There is acceptable agreement between the theoretical and clinically measured wavefront coefficients’ variation for the second and fourth order coefficients’ variations. The planned SE correction (+6.00 D) was better correlated with the theoretical (Δg_2_^0^ = 7.81 µm, ΔSE = 6.02 D) and achieved clinical (Δg_2_^0^ = 7.51 µm, Δ SE = 5.78 D) LD/HD defocus variation than with the Zernike defocus variations (theoretical: Δz_2_^0^ = 9.72 µm, Δ SE = 7.48 D, clinical: Δz_2_^0^ = 9.05 µm, Δ SE = 6.97 D), which tend to overestimate the magnitude of the measured defocus variation.

Postoperatively, uncorrected distance visual acuity with this operated eye was 20/15. The equivalent low degree SE was -1.25 D from the Zernike decomposition (g_2_^0^ = 1.625 µm, 6 mm pupil), and +0.07 D from the LD/HD decomposition (z_2_^0^ = −0.091 µm, 6 mm pupil). The PSF was computed for the combination of fourth and sixth degree wavefront error (WFE) for the Zernike and LD/HD (6 mm pupil). The simulation of Snellen chart using convolutional techniques revealed optotypes identifiable for a resolution equivalent to a 20/50 visual acuity when the eye is corrected for the z_2_^0^ defocus, and to a 20/15 visual acuity when the eye is corrected for the g_2_^0^ defocus (Fig. A2).

### Case 2

A custom aspheric LASIK was performed to compensate for presbyopia in the nondominant eye of a patient with slight hyperopia (preoperative SE = +0.50 D). A custom aspheric correction of +3.00 D with a variation in asphericity of ΔQ = −0.6 was planned to induce a paraxial myopic error and an increase in corneal prolateness, in order to gradually reduce the local vergence of the cornea and increase the depth of focus.^19^^,^^21^ The mean values of the pre and postoperative 1 mm central keratometry and the anterior corneal asphericities were R_0_ = 8.10 mm, R_1_ = 7.60 mm, Q_0_ = −0.49, Q_1_ = −1.43. Figure A1 allows to compare the theoretical versus measured variations for the second, fourth, and sixth order coefficients of the corresponding Zernike versus LD/HD modes (6 mm pupil). There is acceptable agreement between the theoretical and clinically measured wavefront coefficients’ variation for the second and fourth order coefficients’ variations. The theoretical (Δg_2_^0^ = 3.43 µm, Δ SE = 3.00 D) and clinically measured (Δg_2_^0^ = 3.90 µm, Δ SE = 3.34 D) variation in LD/HD second degree coefficient were close to the planned SE correction (Δ SE = 3.00 D). Conversely, the Zernike theoretical and clinically measured variation in z_2_^0^ were largely underestimating the planned SE correction (theoretical SE change = 1.36 D, measured SE change = 0.39 D).

Postoperatively, uncorrected distance visual acuity was 20/60, and best corrected visual acuity was 20/15 with SE = −2.50 D. The predicted low degree SE was −0.14 D (z_2_^0^ = 0.182 µm) from the Zernike decomposition and −3.09 D from the LD/HD decomposition (g_2_^0^ = 4.023 µm). The simulation of Snellen chart reveals optotypes identifiable for a resolution equivalent to a 20/40 visual acuity when the eye is corrected for the z_2_^0^ defocus, and to a 20/15 visual acuity when the eye is corrected for the g_2_^0^ defocus (Fig. A2).
